# Associations Between Positive and Negative Affect and the Way People Perceive Their Health Goals

**DOI:** 10.3389/fpsyg.2020.00334

**Published:** 2020-03-03

**Authors:** Ekaterina Plys, Olivier Desrichard

**Affiliations:** Health Psychology Research Group, Faculty of Psychology and Education Sciences, University of Geneva, Geneva, Switzerland

**Keywords:** health goals, positive affect, negative affect, goal perception, state and trait affect, social judgment bias, goals and means

## Abstract

**Objective:**

Why are people who suffer from depressive symptoms or chronic negative mood less likely to adopt a healthy lifestyle? We postulated that adoption of health goals (HGs) and health behaviors is impeded by negative affect (NA) and facilitated by positive affect (PA). Our aim was to assess the associations between NA and PA, measured as a state and as a trait, and perceptions of HGs and related means. In our studies we tested the relationship between perceptions of HGs and affect measured as a state and as a trait.

**Methods:**

Participants in three online studies were asked to choose and evaluate a health goal (Studies 1–3) or a health goal and related means (Study 3). In Study 1 we used the personal project analysis to assess 10 dimensions of HGs, inter-goal interference, and inter-goal facilitation; in Studies 2 and 3 we used a specially designed questionnaire to assess the difficulty, attainability, controllability, and congruency with self-identity of HGs and related means. We used the Positive and Negative Affect Schedule to measure trait and state affect and the NEO PI-R to measure neuroticism and extraversion.

**Results:**

Participants perceived their HGs and related means in mood-congruent ways. High NA participants perceived their HGs to be less controllable, less attainable, more difficult, and less congruent with their self-identity. They also perceived their related means to be more difficult and less congruent with their self-identity. In contrast, high PA participants perceived their HGs and related means to be more attainable and more congruent with their self-identity, and they evaluated their related means as less difficult. In addition, our results suggest that state affect is better associated with perceptions of HGs than trait affect.

**Conclusion:**

The adoption and attainment of HGs is likely to be facilitated by PA but impeded by NA. PA and NA may also impact the adoption and maintenance of healthy lifestyles. These results help provide a better understanding of the reasons why people with depression or negative mood adhere to behaviors that compromise their health.

## Introduction

Nowadays, the most significant causes of death in developed countries are related to non-communicable diseases and can be prevented by health behaviors (e.g. Khaw et al., 2008). However, many people do not follow healthy lifestyles. One reason for this may be related to negative affect, as health-compromising behaviors are correlated with depressive symptoms and negative mood, and the number of health-compromising behaviors increases with the severity of depression (Allgöwer et al., 2001; Bonnet et al., 2005; Harrington et al., 2009; Vangeli et al., 2010; Taniguchi et al., 2014; Ferrer et al., 2016). However, the reasons why depressive symptoms and negative mood are correlated with health-compromising behaviors remain unclear. The present research investigated one possible factor in this relationship through a series of studies based on the premise that behaviors are largely guided by goals and that goals are likely to be perceived differently depending on a person’s affective state. If this is the case, such differences in perception could be expected to impact the adoption and pursuit of health goals (HGs). Three studies examining the influence of negative affect (NA) and positive affect (PA) on perceptions of HGs and HG-related means were conducted to test this hypothesis. We expected NA to lead to perceptions that would hinder the adoption and pursuit of HGs and related means, and we expected PA to lead to perceptions that would promote the adoption and pursuit of HGs and related means. Furthermore, because affect can be viewed in terms of a stable trait or a variable state, we tested the influence of both trait affect and state affect in order to assess which has more influence on perceptions of HGs.

A goal is ‘a desirable future state of affairs one intends to attain through action’ (Kruglanski, 1996, p. 600). Nevertheless, adopting a goal is a complex process that includes appraising aspects such as the goal’s difficulty, controllability, etc. Hence, individuals are more likely to adopt and pursue a goal if they believe they can control it, see it as congruent with their personal values and identity, feel it is important and attainable, and do not expect conflicts with other important goals (Eiser and Gentle, 1988; Gebhardt, 2006; Rothermund, 2006; Moskowitz, 2012; Mann et al., 2013). These general characteristics apply to all types of goals, including HGs, which are goals individuals set themselves in order to maintain or improve their health. Research into HGs has shown that people tend to prefer easy goals. For example, when Miller et al. (2012) asked individuals to change their eating habits, participants assigned to a difficult goal showed less commitment than participants given an easy goal. In addition, HGs cannot be attained without actions or HG-related means. These actions are important when it comes to evaluating a goal and can influence whether or not it is adopted. For example, people perceive a goal as easier and more attainable if it can be pursued through a variety of means (Shah and Kruglanski, 2000).

The literature shows that people prefer goals that are relatively easy (few barriers impeding HG-related means), controllable, and consistent with their identity. However, such evaluations are malleable. There is evidence that affective states can influence judgments and perceptions of goals. Affect is both a trait and a state, and it can have one of two valences, positive or negative. PA is characterized by joy and high levels of energy, concentration, enthusiasm, and alertness, whereas NA is characterized by distress, anger, contempt, and nervousness or fear (Watson et al., 1988). Trait affect describes a stable tendency to respond to an event with either positive or negative emotions, whereas state affect describes an individual’s affect at a particular moment. Individuals with high trait NA are more prone to NA and tend to worry excessively about errors and threats, and they may also be sensitive to even minimal stressors. Conversely, people with high trait PA are more prone to PA and tend to be more enthusiastic and confident (Watson and Clark, 1984; Watson, 2002). Trait NA and PA are correlated with neuroticism and extraversion, respectively, and these are two personality traits that also reflect the tendency to react negatively or positively to life events. It is interesting to compare the impact of state and trait affect on perceptions of HGs to identify which one has more influence on the perceptions of HGs. This understanding can be important for professionals who work on the promotion of health behaviors. As state affect is changeable and depends on current context, some strategies of emotional regulation can be used to diminish its negative effects. Trait affect is more stable, and it thus allows us to make long-term predictions about how people perceive theirs HGs. However, modification of this personality trait can be a more difficult and time-consuming process.

There is a large body of evidence that shows that affective states influence judgment. According to the mood-congruence effect (Bower, 1981), people make judgments in congruency with their mood, with positive moods leading to more positive judgments about a situation or an object and negative moods leading to more negative judgments. Similarly, according to the Feelings-as-information theory (Schwarz and Clore, 1983; Schwarz, 2012), people refer to their affect when making judgments, and generally make judgments in congruency with their current affect.

Several experimental and correlational studies examining the influence of state and trait affect on a wide variety of targets have reported congruency between mood and judgment. For example, people report greater satisfaction with life on sunny days than on rainy days (Schwarz and Clore, 1983), and, compared with people in a positive mood, people in a negative mood evaluate tasks to be more difficult and demanding, feel they are less likely to succeed, and consider themselves less able to perform a given task (Gendolla, 2000; Gendolla et al., 2001). Eaton and Bradley (2008) found that participants with high trait NA perceived problematic situations as more stressful than those with low trait NA. Similarly, Lerner and Keltner (2000) found that perceptions of risk were positively predicted by trait fear and negatively predicted by trait anger. Studies have also shown that PA and optimism can represent a supplementary resource of coping with difficulties that emerge (Aspinwall, 1998; Aspinwall and Staudinger, 2003). Indeed, PA and optimism facilitate thinking by making people more flexible and open to new information (Estrada et al., 1997; Isen, 2003) and helping them consider alternative ways to solve problems (Isen et al., 1987). It is, thus, possible that people with high PA can perceive their HGs as less difficult and more attainable, as they can consider more possibilities to achieve their goals.

The literature thus shows that judgment is impacted by both state affect and trait affect. Nevertheless, the two types of affect may not be equal predictors of judgment. The Feelings-as-information model (Schwarz and Clore, 1983; Schwarz, 2012) suggests that people rely on their current affect when making a judgment. Although trait affect shows a person’s general tendency to experience either positive or negative moods and emotions, affective states are transient. In other words, people with high trait PA sometimes experience negative moods and people with high trait NA sometimes experience positive moods.

To summarize, although existing research shows that affective states can influence judgment, few studies have investigated the impact of affective states on the perception of goals, especially HGs. In fact, to the best of our knowledge, only perceptions of controllability and the likelihood of attaining approach and avoidance goals have been studied. Dickson et al. (2011) found that, compared with people who had never been depressed, clinically depressed people felt they had less control over their goals, were less likely to attain their approach goals, and were more likely to attain their avoidance goals. Dickson and MacLeod (2006) reported a similar pattern of results following a study of adolescents with depressive symptoms. Unfortunately, other evaluative dimensions, such as a goal’s congruency with self-identity and goal difficulty, which are very important for the adoption and pursuit of HGs, were not studied. In addition, little is known about the impact of PA on perceptions of HGs in the general population or about the impact of affective states on HG-related means. As a first step toward remedying this situation, we carried out three studies into the impact of NA and PA on perceptions of HGs and HG-related means. Study 1 tested the relationship between trait NA and trait PA and perceptions of 10 evaluative dimensions of HGs, inter-goal interference, and inter-goal facilitation. We hypothesized that higher trait NA would be associated with more pessimistic perceptions of HGs, inter-goal interference, and inter-goal facilitation (e.g. goals would be perceived as more difficult or less attainable), whereas PA would be associated with more optimistic perceptions. Studies 2 and 3 built on Study 1 and focused on four dimensions of perceptions of HGs: difficulty, attainability, controllability, and congruency with self-identity. In Study 2, we tested the relationship between both state and trait affect and perceptions of HGs. Our predictions were similar to those for Study 1, that is, we expected higher NA to be linked to higher negative perceptions of HGs and higher PA to be linked to higher positive perceptions. We also expected state affect to be a better associated with perceptions of HGs than trait affect. Finally, Study 3 was designed to replicate Study 2 and to examine the relations between state NA and state PA and perceptions of both HGs and related means. We expected higher NA to be associated with more pessimistic perceptions of HGs and related means and higher PA to be associated with more optimistic perceptions.

## Study 1

In Study 1, we explored the link between trait NA and trait PA on perceptions of HGs, inter-goal interference, and inter-goal facilitation. We hypothesized that higher NA would be associated with perceptions that goals are less controllable, less attainable, less important, and less enjoyable to work toward and that it would also lead to less positive evaluations of progress made, congruency with self-identity and with important personal values, and inter-goal facilitation. We also postulated that higher NA would be associated with higher perceptions of HG difficulty, HG-related stress, challenge, and inter-goal interference. We expected to obtain the opposite pattern for PA.

### Materials and Methods

#### Participants

We conducted our study online, recruiting 118 participants via a crowd sourcing platform called Foul factory that recruits adults living in France. The participants were volunteers who participated in exchange for a payment, and they were informed that they could stop the task at any moment. In order to exclude multiple participations by a single person, we inspected the IP addresses in the database, and if the same IP address was presented more than once, we retained the first observation. The same was applied for studies 2 and 3. We excluded from our analyses 19 people who did not have a HG or whose reported HG was uninterpretable (e.g. “good”), and we also excluded two more participants who participated twice. The remaining 97 participants were aged between 19 and 75 years (*M* = 40.04, *SD* = 11.94), and 57.7% of them were women, 55.7% were in employment, and 46.4% were married or had a partner. The 44.3% of the participants had a degree level of education or higher, 25.8% of them had 2 years of higher education, 18.6% were high school graduates, and 11.3% had less than a high school educational level. Their current health status was rated: 11.3% of the participants rated their health state as very good, 35.1% as good, 34% as satisfactory, 16.5% as rather bad, and 3.1% as bad. The HGs reported most frequently were related to exercising (32%), healthy diet and weight loss (25%), preserving one’s health (19%), recovering from an illness or treating an illness (12%), quitting smoking (7%), and other (5%).

To the best of our knowledge, this is the first research paper written on the impact of NA and PA on perceptions of HGs, and thus no reference from a previously obtained effect size has been included in this research. We suppose that the effect size will be between small and medium, which corresponds to Cohen’s *f*^2^ equal to 0.08. The power analysis conducted with G^∗^Power (Faul et al., 2009) indicated that for the multiple regression analysis, the total sample size of 124 people (who have a HG) was needed to detect an effect size between small and medium with 80% power and alpha at 0.05. We used the same estimations of sample size for studies 2 and 3. This and the other two studies described in this paper were approved by the Ethics Committee of the Faculty of Psychology and Educational Sciences of the University of Geneva. Our studies were carried out in accordance with the faculty’s code of ethics and university’s guidelines on research integrity. All the participants gave written informed consent.

#### Measures

##### Assessment of trait NA and PA

We measured trait NA and PA via the French version of the Positive and Negative Affect Schedule (PANAS; Watson et al., 1988), stipulating “in general” as the time instruction; the participants were asked to evaluate how they feel in general most of the time. The PANAS contained 20 adjectives describing feelings and emotions that participants had to rate on a 5-point scale (1 = very slightly or not at all; 5 = extremely). Mean scores (and standard deviations) were *M* = 34.33 (*SD* = 5.31) for PA and *M* = 24.55 (*SD* = 7.08) for NA. Reliability coefficients were acceptable for the PA scale (Cronbach’s alpha = 0.79) and good for the NA scale (Cronbach’s alpha = 0.87).

##### Assessment of HGs

We assessed HGs via a modified version of the Personal project analysis (PPA; Little, 1983), focusing on ten dimensions: goal importance, goal difficulty, enjoyment of working on the goal, perceived controllability, challenges relating to goal realization, stress relating to goal realization, goal attainability, goal congruency with self-identity, goal congruency with important values in life, and evaluation of progress made. We also assessed inter-goal facilitation and interference. In our study we were interested in subjective evaluations of the participants’ HGs, and we therefore excluded the five resting PPA dimensions that did not meet this requirement. Thus, several dimensions were rather objective evaluations (whether the goal was chosen by the participant or imposed by someone or whether the amount of time spent working on the goal was sufficient) related to perceptions of other people (perceptions of the goal by significant others) or to communication with others (to what extent the participant informed his/her family and friends about the fact of pursuing the goal). Absorption was excluded because this dimension refers to the perception of personal involvement in the goal striving and not the goal *per se*. Participants evaluated the PPA dimension on 11-point scales, from 0 (lowest level of the dimension, e.g. the goal is not at all challenging) to 10 (highest level of the dimension, e.g. the goal is very challenging). We measured inter-goal interference and inter-goal facilitation by asking participants to write down another three important personal goals unrelated to health and to estimate the extent to which their HG interfered with or facilitated the attainment of these three goals and the extent to which they interfered with or facilitated the attainment of the HG. The participants responded using an 11-point scale, where 10 corresponded to high inter-goal facilitation or interference (e.g. the goal facilitate a lot progress to other goals) and 0 corresponded to low facilitation or interference (e.g. the goal has no influence on the progress to other goals). In the data analysis, we used the mean scores of responses for inter-goal facilitation and inter-goal interference. A pilot study showed that the instructions, items, and evaluation scales of our French version of the PPA were clear. We also checked whether people who had HGs and people who did not have HGs differ in positive and negative affect. The participants who reported HGs had a mean NA equal to 24.55 (*SD* = 7.08) and mean PA equal to 34.33 (*SD* = 5.31). Those who did not reported HGs had a mean NA of 24.19 (*SD* = 7.31) and mean PA equal to 33.06 (*SD* = 8.59).

For exploratory purposes, we also assessed four health behaviors: smoking, alcohol consumption, fruit and vegetable consumption, and physical activity. We do not report these results here because they are beyond the scope of the present paper.

#### Procedure

Participants were instructed to write down a HG and another three important goals. They then evaluated their HG with respect to 10 dimensions of the PPA and inter-goal facilitation and inter-goal interference. Participants who did not have a HG were asked to report and evaluate an important goal unrelated to health (but their answers were not analyzed). Finally, participants completed the PANAS and a socio-demographic questionnaire. Participants were thanked and those who completed all the questionnaires were paid €5.

### Data Analysis

In all three studies, we tested our hypotheses by conducting the multiple regression analysis. Since for each HG evaluative dimension a separate multiple regression was calculated, we applied a Holm correction for multiple testing to control for error rates (Aickin and Gensler, 1996). Studies have shown that health behaviors, such as smoking, alcohol consumption, and physical activity, change with age (Piazza et al., 2001; Rochelle, 2015), differ in men and women (Allgöwer et al., 2001; Bonnet et al., 2005; Raffaelli et al., 2013; Lovell et al., 2015), differ in people who live alone or with a partner (Lee et al., 2004; Eng et al., 2005; Osler et al., 2008), and differ in those who rate their health status as rather good or rather bad (Lu et al., 2002; Kim and Cho, 2010). For this reason, the control variables of the present research were age, sex, partnership status and perceived health. In Study 1, two more control variables were used, namely, level of education and employment, but these variables were omitted in studies 2 and 3. All the statistical analyses were conducted using the IBM SPSS Statistics 23.

### Results

First, we conducted a correlational analysis for the PPA dimensions, positive and negative affect, and control variables (see Table 1 for details). Most of the correlations between HG evaluative dimensions were small or medium, suggesting that the PPA dimension represented independent constructs. The only high correlations (exceeded 0.5) were observed between perceptions of congruency with self-identity and congruency with values (*r* = 0.679), between perceptions of attainability and congruency with values (*r* = 0.537), attainability and progress made (*r* = 0.512), and difficulty and challenge (*r* = 0.5). Despite these correlations indicating rather strong associations between variables, they cannot be considered as interchangeable. NA was positively correlated with stress related to goal attainment and inter-goal conflicts and negatively correlated with HG attainability and congruency with self-identity and values. PA was positively correlated with enjoyment working on the HG, HG attainability, progress made, and congruency with self-identity and negatively correlated with NA, HG difficulty, stress, and challenge.

**TABLE 1:** Correlations between the evaluative dimensions of health goals, positive and negative affect, and age, sex, perceived health, whether the participant lived alone or with a partner, educational level, and employment for the Study 1 (*N* = 99).

	**1**	**2**	**3**	**4**	**5**	**6**	**7**	**8**	**9**	**10**	**11**	**12**	**13**	**14**	**15**	**16**
1. Importance	–															
2. Enjoyment	0.409**	–														
3. Difficulty	−0.203*	−0.246*	–													
4. Control	0.132	0.375**	−0.278**	–												
5. Stress	0.027	–0.076	0.405**	−0.357**	–											
6. Attainability	0.179	0.422**	−0.360**	0.470**	−0.247*	–										
7. Congruency with identity	0.292**	0.288**	–0.185	0.287**	−0.259*	0.455**	–									
8. Congruency with values	0.265**	0.280**	–0.054	0.308**	–0.103	0.537**	0.679**	–								
9. Progress	0.363**	0.575**	−0.461**	0.413**	−0.204*	0.512**	0.376**	0.347**	–							
10. Challenge	–0.056	0.088	0.500**	–0.023	0.290**	–0.083	–0.003	0.078	–0.150	–						
11. Conflicts	0.138	0.123	0.113	−0.210*	0.345**	−0.230*	–0.054	–0.137	–0.074	0.240*	–					
12. Facilitation	0.222*	–0.142	−0.230*	0.138	0.129	0.169	0.070	0.130	0.281**	–0.002	0.410**	–				
13. NA	–0.032	–0.058	0.095	–0.132	0.418**	−0.272**	−0.378**	−0.292**	–0.172	0.189	0.215*	0.174	–			
14. PA	0.120	0.201*	−0.226*	0.092	−0.219*	0.210*	0.232*	0.145	0.286**	−0.278**	–0.070	0.104	−0.384**	–		
15. Age	0.063	0.115	−0.259*	0.117	–0.026	–0.005	0.017	0.017	0.125	–0.189	–0.009	0.026	–0.124	–0.009	–	
16. Perceived health	–0.059	0.194	−0.238*	0.263**	−0.464**	0.207*	0.264**	0.204*	0.317**	−0.245*	−0.347**	0.067	−0.239*	0.428**	–0.028	–
17. Educational level	–0.070	–0.142	0.240*	–0.036	0.009	0.018	0.004	0.033	–0.055	0.016	–0.031	–0.108	–0.066	0.030	−0.315**	0.094

*p ≤ 0.05; **p ≤ 0.01.

The main analyses showed that, in line with our hypotheses, higher NA was associated with lower congruency with self-identity and higher stress. None of the results for PA were significant. Table 2 shows the effects of NA and PA on each dimension of the PPA together with the mean and standard deviation for each dimension.

**TABLE 2:** Unstandardized regression coefficients [and 95% confidence intervals] for the relationship between the dimensions of the PPA and trait NA and PA controlled by age, sex, perceived health, level of education, employment, and whether the participant lived with a partner or alone (Study 1, *N* = 99).

**PPA dimensions**	**NA**	**PA**	***M* (*SD*)**
Importance	−0.005 [−0.059, 0.049]	0.048 [−0.027, 0.124]	8.2 (1.74)
Enjoyment	0.030 [−0.051, 0.112]	0.070 [−0.044, 0.184]	5.59 (2.82)
Difficulty	−0.002 [−0.076, 0.073]	−0.073 [−0.177, 0.032]	6.44 (2.52)
Controllability	−0.020 [−0.094, 0.054]	−0.024 [−0.127, 0.079]	5.61 (2.33)
Stress	0.155* [0.074, 0.237]	0.058 [−0.056, 0.173]	5.2 (3)
Attainability	−0.059 [−0.124, 0.005]	0.024 [−0.066, 0.114]	6.29 (2.09)
Congruency with self-identity	−0.105* [−0.169, −0.041]	0.008 [−0.082, 0.097]	6.99 (2.18)
Congruency with values	−0.072 [−0.131, −0.013]	−0.015 [−0.097, 0.068]	7.62 (1.95)
Progress	−0.007 [−0.081, 0.066]	0.074 [−0.029, 0.177]	5.79 (2.52)
Challenge	0.027 [−0.047, 0.100]	−0.088 [−0.191, 0.015]	7.5 (2.4)
Inter-goal interference	0.066 [−0.012, 0.144]	0.075 [−0.034, 0.184]	2.5 (2.54)
Inter-goal facilitation	0.097 [0.012, 0.182]	0.081 [−0.039, 0.201]	4.86 (2.7)

*M* (*SD*), mean (and standard deviation) for each dimension of the PPA; NA, negative affect; PA, positive affect. *Statistically significant results after Holm correction.

### Discussion

Study 1 explored the relationship between trait NA and PA and perceptions of HGs. In line with our predictions, people with high NA perceived their HGs as more stressful and less congruent with their identity. However, NA had a more limited impact than expected, as we did not find links between NA and most of the evaluative dimensions of perception of HGs (importance, enjoyment, difficulty, controllability, attainability, congruency with values, progress, challenge, and inter-goal facilitation and interference). In addition, PA had no effect on perceptions of any of the evaluative dimensions. Thus, we failed to replicate the results of previous studies, which have reported mood-congruent judgments. Our results also contrasted with Dickson et al. (2011) and Dickson and MacLeod (2006), who found that perceptions of goal controllability were affected by clinical depression and depressive symptoms.

We may have failed to replicate Dickson et al. (2011) and Dickson and MacLeod (2006) results; their studies focused on participants who were suffering from clinical or sub-clinical depression, which, in addition to negative mood, is characterized by other factors (lack of energy, indecisiveness, and diminished ability to think or concentrate; *DSM-V-TR*, 5th ed., rev.; American Psychiatric Association [APA], 2013) that may independently influence perceptions of goals. Studies comparing clinically depressed and non-depressed participants are needed to test this hypothesis.

Second, the lack of significant links between affect and HGs may be due to the fact that our perceptions of HG measures included only one question per dimension. Multi-item scales have been shown to have much greater predictive validity than single-item measures (Diamantopoulos et al., 2012). Moreover, the performance of single-item measures depends considerably on the construct measured and can be affected by responses given to previous items (carry-over effect). To overcome this limitation in subsequent studies, we created a special questionnaire with three questions for each evaluative dimension.

In addition, the way we measured NA and PA may also have contributed to our failure to find significant impacts of affect. Although there is evidence that both state affect and trait affect influence judgment, we suggest that state affect will be better associated with HG evaluative dimensions. According to the Feelings-as-information model (Schwarz and Clore, 1983; Schwarz, 2012), people use their current affect when making a judgment. In Study 2, we tested the link between perceptions of HGs and both state affect and trait affect. We used the PANAS to measure state PA and NA and the NEO PI-R questionnaire (Goldberg et al., 2006) to measure extraversion and neuroticism. Although both state and trait affect can be assessed using the PANAS, we decided to use different questionnaires. The reason was that neuroticism and extraversion are strongly linked to trait NA and trait PA, respectively (Watson et al., 1988). Secondly, we wanted to avoid any potential bias in our measures that may have occurred due to the administration of the same questionnaire twice within a period of minutes in order to measure trait affect and then state affect. The PANAS was validated with different temporal instructions that allowed us to assess state affect (the participants are asked to assess his/her momentary affect with the instructions “right now”) and trait affect (the participants are asked to assess their affect in general, Watson et al., 1988). Moreover, Watson and Walker (1996) studied temporal validity of the PANAS by assessing the affect of the participants within 7 years and found that the questionnaire showed good stability (the rank order analysis correlations were of 0.43 for NA and of 0.42 for PA). The relationship between affect and personality traits has been discussed by many authors. Tellegen (1985) suggested that neuroticism and extraversion are related to trait NA and PA, respectively, because these personality traits reflect stable tendencies to experience NA and PA. Watson and Clark (1984) added that trait NA reflects stable individual differences in the level of experienced negative emotions and in self-concept. Individuals with a high level of negative affectivity are more likely to be distressed, upset, and to have a negative view of themselves, whereas the people with low negative affectivity are “relatively content and secure and satisfied with themselves.” Moreover, Meyer and Shack (1989) found in their study significant correlations between trait PA and extraversion at 0.66 and between PA and neuroticism at −0.17. Similarly, NA correlated with neuroticism at *r* = 0.63 and at −0.22 with extraversion.

Finally, the lack of statistically significant results could be related to the fact that this study was underpowered. A sample size of 124 people is needed in order to detect the expected effect size *f*^2^ = 0.08. After data cleaning and exclusion of the participants who did not have HG, our sample included 97 participants. For our further studies, we need recruit more participants in order to achieve adequate power.

In Study 2, we reduced the number of evaluative dimensions of HGs and used only difficulty, attainability, controllability, and congruency with self-identity. When making choices of evaluative dimensions we were guided by many reasons. Firstly, the new HG perceptions questionnaire included three questions for each dimension, and we were restricted in terms of questionnaire length. Secondly, the selected dimensions had to be potentially influenced by affect. Thus, the goal importance had to be excluded, as Meyer et al. (2004) found that this dimension was not influenced by affect. Moreover, the chosen dimensions had to be of great importance for the adoption and pursuit of HGs. Finally, in order to be consistent, we selected the dimensions among those used in Study 1. We retained goal difficulty because this parameter is positively related to effort mobilization, goal commitment, and performance (Klein et al., 2013; Locke and Latham, 2013). Attainability is also positively related to goal commitment (Shah and Higgins, 1997) and is crucial for adopting goals because people are more likely to adopt attainable goals (Moskowitz, 2012). Perceived control over goal attainment is strongly related to effort mobilization and persistence in pursuing goals (Rothermund, 2006). Finally, congruency with self-identity is crucial for adopting HGs because people are more likely to adopt goals that are congruent with their identity (Gebhardt, 2006). We removed the other dimensions (including perceived stress, even though we obtained a significant result for this dimension in Study 1) because they did not appear to be central aspects of goal adoption and pursuit processes.

## Study 2

Study 2 tested the relationship between state affect and trait affect with four dimensions of perceptions of HGs. We hypothesized that state and trait NA would be negatively associated with perceptions of HG attainability, controllability, and congruency with self-identity and would be positively associated with HG difficulty. We expected the reverse to be true for state and trait PA. We also expected state affect to be a better associated with perceptions of HGs than trait affect.

### Materials and Methods

#### Participants

We recruited participants via an online platform. From the initial sample of 202 participants, we excluded 17 participants who did not report a HG. Hence, our analyses were based on the responses provided by the 185 participants who reported a HG. The participants were aged between 18 and 75 years (*M* = 40.67, *SD* = 11.85), and 63.2% of them were women, and 66.3% were married or in a relationship. An evaluation of their perceived health was provided: 10.3% estimated their health as very good, 44.6% as good, 32.6% as satisfactory, 12% as rather bad, and 0.5% as bad. The most frequently chosen HGs were related to weight loss or maintenance of healthy weight (22.8%), healthy eating and exercising (16.8%), and practice a regular physical activity (15.8%). Other reported HGs were related to stopping or reducing smoking (9.2%) or alcohol consumption (2.7%), improving diet (8.7%), adopting healthy habits to live longer (7.1%) or a proper daily hygiene (6%), improving health (3.8%), healing a disease (3.3%), preventing risk of relapse (0.5%), and other (1.1%).

#### Measures

##### Assessment of HGs

The HG perceptions questionnaire had two parts. The first part asked participants to choose a HG from a list of 12 HGs drawn up on the basis of the HGs reported by participants in Study 1. The list included several goals: stop or reduce smoking, eat healthy and exercise, stop or reduce alcohol consumption, improve diet, practice a regular physical activity, lose weight or maintain a manageable weight, adopt healthy habits to live longer, heal a disease, improve my health, adopt proper daily hygiene, prevent risk of relapse, and avoid serious diseases. Participants whose HG was different from those included in the list could write down a specific HG in a separate field. Part two consisted of a 12-item questionnaire evaluating the four evaluative dimensions of HGs: difficulty (e.g. *working on my HG requires of me serious efforts*), attainability (e.g. *I believe that I will achieve my goal*), controllability (e.g. *achievement of my HG depends on factors that are beyond my control*), and congruency with self-identity (e.g. *my HG is in coherence with who I am*). The questionnaire included three items for each dimension. Participants were asked to rate each item on a 11-point scale (1 = *somewhat disagree*, 11 = *completely agree*). Participants who had no HG were asked to state an important goal and to evaluate it on the same four dimensions. Two pilot studies carried out to test the questionnaire gave acceptable to good reliability coefficients for difficulty (Cronbach’s alpha = 0.734), attainability (0.885), and controllability (0.816). The reliability coefficient for self-identity was questionable (0.625). Mean scores (and standard deviations) for perceptions of the four dimensions of HGs were *M* = 20.8 (6.28) for difficulty, *M* = 23.22 (5.82) for attainability, *M* = 23.32 (7.17) for controllability, and *M* = 23.54 (5.49) for congruency with self-identity. The mean NA of the participants who reported a HG was 17.04 (*SD* = 6.29), and the mean PA was 30.48 (*SD* = 6.28). The mean NA of the participants who did not have a HG was 16.71 (*SD* = 7.78), while the mean PA was 30 (7.19).

##### Assessment of affect

We assessed state affect with the PANAS (see section “Study 1” for details), stipulating “at the moment” as the time instruction. In this study, the PANAS was administered before the questionnaire on HGs in order to separate the two measures of affect (made by the PANAS and by the NEO PI-R) and avoid potential bias. Means and standard deviations for NA and PA were 15.16 (6.33) and 30.92 (5.99), respectively. The reliability coefficients were excellent for NA (Cronbach’s alpha = 0.913) and good for PA (Cronbach’s alpha = 0.825). Trait NA and PA were assessed via the neuroticism and extraversion sub-scales of the International Personality Item Pool (IPIP) version of the NEO PI-R questionnaire (Goldberg et al., 2006; International Personality Item Pool, 2018). Each sub-scale included 10 items that participants had to rate the statements on the questionnaire on 5-point scales, from 1 = *Very inaccurate* to 5 = *Very accurate*. Total scores for each sub-scale were calculated by summing the scores for the five positively keyed and the five negatively keyed items (after the negatively keyed items had been inversed). Correlations between the 10-item English versions of the neuroticism and extraversion scales and the original NEO PI-R neuroticism and extraversion sub-scales were 0.82 and 0.77, respectively. The IPIP provides French translations for several of the scales’ items; the remaining items were translated and backtranslated by an English native speaker and a French native speaker from our research group. Means and standard deviations were 28.15 (8.01) for neuroticism and 28.68 (7.43) for extraversion. Cronbach’s alphas for both sub-scales were good (0.884 for neuroticism and 0.870 for extraversion).

#### Procedure

Participants filled in the PANAS and then chose and evaluated their HG. Participants who did not have HGs evaluated another important goal, but we did not include their responses in the analyses. Next, they completed the neuroticism and extraversion measures and a socio-demographic questionnaire. Finally, we thanked the participants and paid them €2 each.

### Results

We conducted a correlational analysis between the evaluative dimensions of HGs, measures of state and trait affect, and control variables (see Table 3). Most of the correlations between HG evaluative dimensions were small or medium which indicates that these evaluative dimensions can be view as independent. There were only two strong correlations, and these were between perceptions of attainability and difficulty (*r* = −0.526) and between perceptions of attainability and congruency with self-identity (*r* = 0.690). NA was negatively correlated with congruency with self-identity. PA was positively correlated with attainability of HGs and with congruency with self-identity. Neuroticism was negatively correlated with attainability of HGs and congruency with self-identity and positively correlated with HG difficulty. There were no correlations between extraversion and HG dimensions.

**TABLE 3:** Correlations between the evaluative dimensions of HGs, state and trait affect, age, sex, perceived health, and whether the participant lived alone or with a partner for the Study 2 (*N* = 185).

	**1**	**2**	**3**	**4**	**5**	**6**	**7**	**8**	**9**
1. HG difficulty	–								
2. HG attainability	−0.526**	–							
3. HG controllability	−0.370**	0.402**	–						
4. Congruency with self-identity	−0.422**	0.690**	0.330**	–					
5. NA	0.086	–0.134	–0.079	−0.216**	–				
6. PA	–0.134	0.264**	0.024	0.275**	0.016	–			
7. Neuroticism	0.246**	−0.263**	–0.066	−0.272**	0.449**	−0.153*	–		
8. Extraversion	0.038	0.064	–0.133	0.137	–0.139	0.326**	−0.462**	–	
9. Age	–0.082	0.048	–0.131	0.092	–0.054	0.129	–0.068	0.205**	–
10. Perceived health	−0.479**	0.367**	0.361**	0.251**	–0.088	0.125	−0.207**	–0.093	−0.156*

*p ≤ 0.05; **p ≤ 0.01.

Next, we carried out multiple regression analyses to test separately the effects of state NA and PA and trait NA and PA (measured via neuroticism and extraversion). Results showed that state PA was positively associated with HG attainability and congruency with self-identity, and state NA was negatively associated with congruency with self-identity (see Table 4). In the case of trait affect, neuroticism was positively associated with difficulty. No other results were statistically significant after applying Holm corrections.

**TABLE 4:** Unstandardized regression coefficients [and 95% confidence intervals] for relationship between state and trait NA and PA and perceptions of HGs controlled by age, sex, perceived health, and whether the participant lived alone or with a partner (Study 2, *N* = 185).

	**Difficulty**	**Attainability**	**Controllability**	**Self–identity**
State NA	−0.065 [−0.072, 0.202]	−0.086 [−0.218, 0.045]	−0.020 [−0.187, 0.148]	−0.166* [−0.290, −0.041]
State PA	−0.044 [−0.184, 0.096]	0.201* [0.067, 0.336]	−0.017 [−0.188, 0.155]	0.205* [0.078, 0.333]
Neuroticism	0.155* [0.036, 0.273]	−0.125 [−0.241, −0.008]	−0.039 [−0.187, 0.108]	−0.111 [−0.223, 0.001]
Extraversion	0.108 [−0.019, 0.235]	0.012 [−0.113, 0.137]	−0.117 [−0.275, 0.040]	0.067 [−0.053, 0.187]

NA, negative affect; PA, positive affect. *Statistically significant results after Holm correction.

### Discussion

Study 2 tested the effects of state NA and PA and trait NA and PA on perceptions of HG difficulty, attainability, controllability, and congruency with self-identity. As in Study 1, NA was negatively associated with perceptions of HG congruency with self-identity. This result can be explained by the use of the same questionnaire (the PANAS) in order to assess NA in both studies with the only difference that in Study 1 we applied the time instructions “in general” in order to measure trait NA, while, in Study 2, we applied the time instructions “at the moment,” which assessed state NA. We also found that trait NA, measured using the NEO PI-R neuroticism sub-scale, was negatively associated with the difficulty of HGs. These results are consistent with our hypotheses, according to which people make judgments in congruency with their mood.

In addition, state PA was positively associated with HG attainability and congruency with self-identity, which was also in line with our hypotheses. Participants who were in a positive mood made more optimistic judgments about the attainability of their HGs and their HGs’ congruency with their identity. Neither study displayed an effect of trait PA on perceptions of HGs, which suggests that only state PA impacts these perceptions. This is consistent with the Feelings-as-information theory (Schwarz and Clore, 1983; Schwarz, 2012), according to which people use their current affect when making judgments. Our results also accord with those reported by George (1991); Kluemper et al. (2009), and Mustanski (2007), who found that state was a better predictor of measured outcomes. Nevertheless, our results were insufficient, and we were thus not able to conclude whether state affect is better associated with perceptions of HGs than trait affect. Further studies are needed to answer this question.

In conclusion, Study 2 showed people perceive their HGs in congruence with their affect and that state affect appeared to be better associated with HG perceptions than trait affect. Both findings were in line with our predictions. Nevertheless, our studies provided only a limited number of results. Moreover, in Study 2, we observed effects of affect on attainability and difficulty that we did not observe in Study 1. This may be because the questionnaire used to measure perceptions of HGs in Study 2 was more sensitive than the questionnaire used in Study 1 and therefore revealed more effects. However, this variability meant we needed to try and replicate our results.

We expected the results of our third study to replicate those of Study 2. We focused on the impact of state PA and NA on perceptions of HGs because, according to the Feeling-as-information theory (Schwarz and Clore, 1983; Schwarz, 2012), people use their current affect when making judgments. Study 3 also extended Study 2 by including evaluations of HG-related means. As noted in the introduction, goal-related means are an important part of evaluating goals and may influence their adoption and pursuit. Consequently, if an affective state is associated with perceptions of HG-related means, this will impact whether or not people implement those means and, potentially, whether or not they adopt and pursue HGs or health behaviors. We postulated that affect impacts perceptions of the difficulty, attainability, controllability, and congruency with self-identity of HG-related means in the same way it impacts perceptions of HGs.

## Study 3

Study 3’s aims were to replicate Study 2 and to test the effect of state NA and PA on perceptions of HGs and related means. As in Study 2, we expected high NA to be associated with perceptions that HGs are more difficult, less attainable, less controllable, and less congruent with self-identity. We also expected high NA to have similar effects on perceptions of HG-related means. We expected the opposite pattern of results for PA.

### Materials and Methods

#### Participants

Once again, we carried out our study online, recruiting participants via an online platform. From the initial sample of 211 participants, we excluded 31 participants who did not report HGs or who reported an incongruous means for attaining their HG (e.g. suggesting *sport* as a means for attaining the goal of *eating more healthily*). Hence, our analyses were based on the responses provided by the remaining 180 participants. These participants were aged between 18 and 74 years (*M* = 40.86, *SD* = 12.73), and 63.9% of them were women, and 68.3% were married or had a partner. Our participants evaluated their current health state: 13.9% of them evaluated their health as very good, 38.9% as good, 34.4% as satisfactory, 10.2% as rather bad, and 2.2% as bad. The most of our participants had HGs related to healthy eating and exercising (23.9%), weight loss or maintenance of healthy weight (23.9%), practice a regular physical activity (18.9%), and quitting or reducing smoking (12.2%). Other chosen HGs were related to improving diet (4.4%), adopting healthy habits to live longer (5.6%) or adopting proper daily hygiene (3.3%), healing a disease (2.8%), improving their health (2.2%), preventing risk of relapse (1.1), and other (1.1%).

#### Measures

##### Assessment of perceptions of HGs and related means

We used the perceptions of HGs questionnaire described in Study 2 to evaluate perceptions of HGs. In Study 3, the reliability coefficients for this questionnaire were acceptable to good for difficulty (Cronbach’s’ alpha = 0.768), attainability (0.872), and controllability (0.737), but it was poor for congruency with self-identity (0.579). Means (and standard deviations) for the questionnaire were *M* = 20.17 (6.53) for difficulty, *M* = 24.48 (5.52) for attainability, *M* = 22.83 (6.83) for controllability, and *M* = 24.01 (5.32) for congruency with self-identity.

The questionnaire we used to evaluate HG-related means was inspired by the HG perceptions questionnaire and contained similar questions grouped into the same four dimensions (for example, one of the statements used to assess mean difficulty was *It is not difficult for me to do this activity*). The participants were asked to assess each item on a 11-point scale where 1 = *somewhat disagree* and 11 = *completely agree*. Participants were instructed to write down a means they use to attain their HG and to evaluate its difficulty, attainability, controllability, and congruency with self-identity. Cronbach’s alphas for HG-related means ranged from acceptable to good: 0.733 for difficulty, 0.876 for attainability, 0.812 for controllability, and 0.734 for congruency with self-identity. Means (and standard deviations) for HG-related means were *M* = 15.94 (7.91) for difficulty, *M* = 22.66 (9.07) for attainability, *M* = 21.45 (8.19) for controllability, and *M* = 25.48 (5.91) for congruency with self-identity. Mean NA of the participants who reported a HG was 15.16 (*SD* = 6.33), and their mean PA was 30.92 (*SD* = 5.99). Mean NA of the participants who did not have HG was 17.06 (*SD* = 7.28), and their mean PA was 29.24 (4.7).

##### Assessment of state NA and PA

We assessed state NA and PA via the PANAS, stipulating “at the moment” as the time instruction. Cronbach’s alphas for the PA and NA scales were good (0.864 and 0.882, respectively). Means (and standard deviations) were *M* = 30.48 (6.28) for PA and *M* = 17.04 (6.29) for NA.

For exploratory purposes, we also included measures for anger and sadness, but we have not reported these results here because they are beyond the scope of the present paper.

#### Procedure

Participants were invited to choose and evaluate HGs and related means. They then filled in the PANAS and the socio-demographic questionnaire. Participants who did not have HGs were asked to evaluate an important goal and a related means of their choice, but we did not include their responses in our analyses. Participants who completed all the questionnaires were paid €2.

### Results

The correlational analysis showed that several dimensions of HG and means perceptions were strongly associated. Thus, controllability of HGs was correlated with congruency with self-identity at 0.625, perceptions of means attainability was correlated with means difficulty at 0.723, and congruency of means with self-identity was correlated with congruency of HG with self-identity at 0.553. The other evaluative dimensions of HGs and means showed moderate or low correlation and were thus independent constructs. The results of the correlational analysis are presented in Table 5. NA and PA were correlated with evaluative dimensions of HG and means. Thus, NA was positively correlated with HG difficulty and negatively correlated with HG and mean attainability, HG controllability, and HG and means congruency with self-identity. PA was negatively correlated with HG and means difficulty, and it was positively correlated with HG and means attainability as well as HG and means congruency with self-identity.

**TABLE 5:** Correlations between the evaluative dimensions of HGs, related means, state affect, age, sex, perceived health, and whether the participant lived alone or with a partner (Study 3, *N* = 180).

	**1**	**2**	**3**	**4**	**5**	**6**	**7**	**8**	**9**	**10**	**11**
1. HG difficulty	–										
2. HG attainability	−0.483**	–									
3. HG controllability	–0.146	0.305**	–								
4. HG congruency with self-identity	−0.482**	0.625**	0.193**	–							
5. Means difficulty	0.216**	−0.236**	–0.071	−0.280**	–						
6. Means attainability	–0.092	0.238**	0.085	0.296**	−0.723**	–					
7. Means controllability	0.053	0.102	0.329**	0.128	−0.322**	0.405**	–				
8. Means congruency with self-identity	−0.206**	0.427**	0.249**	0.553**	−0.489**	0.462**	0.194**	–			
9. NA	0.237**	−0.410**	−0.200**	−0.284**	0.133	−0.180*	–0.132	−0.291**	–		
10. PA	−0.160*	0.293**	–0.064	0.273**	−0.248**	0.312**	0.064	0.274**	−0.177*	–	
11. Age	–0.124	0.094	–0.046	0.101	−0.221**	0.156*	0.031	0.124	−0.191*	0.109	–
12. Perceived health	−0.461**	0.331**	0.262**	0.318**	−0.278**	0.215**	0.092	0.264**	−0.187*	0.136	–0.040

HG, health goal; NA, negative affect; PA, positive affect. *p ≤ 0.05; **p ≤ 0.01.

The regression analyses showed that NA and PA influenced perceptions of both HGs and HG-related means. NA negatively predicted perceptions of HG attainability, controllability, and congruency with self-identity, and negatively predicted perceptions of the congruency of HG-related means with self-identity. In addition, PA positively predicted perceptions of the attainability and congruency with self-identity of both HGs and HG-related means and negatively predicted perceptions of the difficulty of HG-related means (see Table 6 for details).

**TABLE 6:** Unstandardized regression coefficients [and 95% confidence intervals] for relationship between affect and perceptions of HGs and HG-related means controlled by age, sex, perceived health, and whether a participant lived alone or with a partner (Study 3, *N* = 180).

**Predictor**	**Difficulty**	**Attainability**	**Controllability**	**Self-identity**
	**HGs**			

NA	0.128 [−0.013, 0.269]	−0.291* [−0.409, −0.174]	−0.210* [-0.371, −0.049]	−0.163* [−0.282, −0.043]
PA	−0.053 [−0.196, 0.089]	0.189* [0.071, 0.308]	−0.141 [−0.303, 0.022]	0.171* [0.050, 0.292]

	**HG-related means**			

NA	0.026 [−0.152, 0.204]	−0.131 [−0.336, 0.074]	−0.154 [−0.356, 0.048]	−0.175* [−0.304, -0.046]
PA	−0.263* [−0.442, −0.083]	0.437* [0.230, 0.644]	0.076 [−0.128, 0.280]	0.228* [0.097, 0.358]

NA, negative affect; PA, positive affect. *Statistically significant results after Holm correction.

### Discussion

In Study 3, we tested the relationship between state NA and PA and perceptions of HGs and related means. Results replicated those of Study 2, as state NA was negatively associated with congruency of HGs with self-identity, whereas state PA was positively associated with perceptions of HG attainability and congruency with self-identity. We found similar effects of state NA and PA on perceptions of HG-related means. Hence, the links of current NA and PA and these dimensions of HGs appeared to be stable and to apply to perceptions of HG-related means.

In addition, state PA was negatively associated with perceived difficulty of HG-related means. This was in line with our hypothesis and with results reported by Gendolla (2000) and Gendolla et al. (2001), who found that people in a positive mood perceived their tasks as less difficult than those in a negative mood. Finally, we also obtained an association between state NA and perceptions of HG controllability and attainability. Although this result was consistent with our hypotheses, we did not observe this effect in Studies 1 or 2. Further work is needed to clarify the effects of NA and PA on perceptions of HG controllability and attainability.

## Meta-Analysis of the Results

In order to summarize our findings, we conducted a meta-analysis, but we included only the effects of NA and PA on perceptions of HGs. We carried out this meta-analysis using the Exploratory Software for Confidence Intervals (ESCI) developed by Cumming (2012) and applied the random effects model, as recommended by Cumming.

**FIGURE 1:**
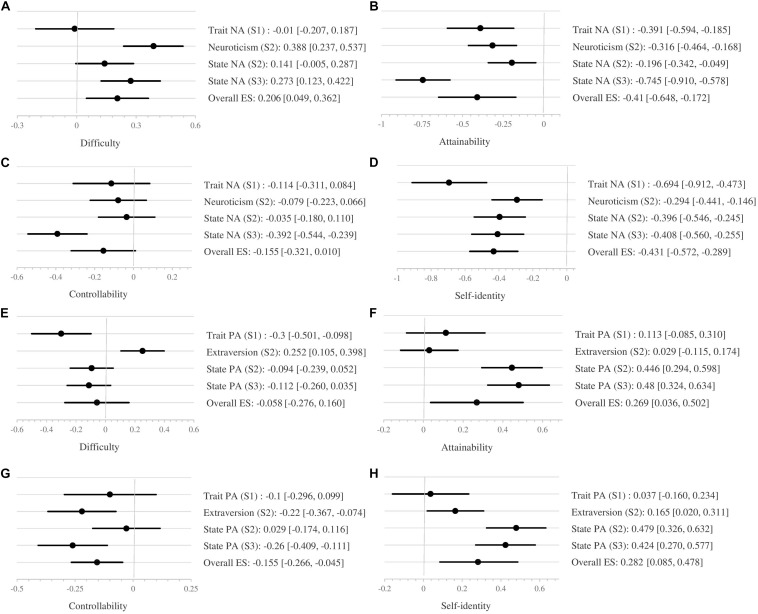
**(A–H)** Forest plots of the influence of NA and PA on perceptions of HGs. S1, study 1; S2, study 2; S3, study 3.

The meta-analysis gave overall effect sizes (Cohen’s *d*) of between 0.058 and 0.431. The dimensions most influenced by affect were HG difficulty, attainability, and congruency with self-identity with effect sizes that were between small and medium, as defined by Cohen (1988). Difficulty was influenced only by NA, whereas attainability and congruency with self-identity were influenced by both NA and PA. The effects of NA and PA on perceptions of controllability (for both NA and PA) and difficulty (PA only) were less pronounced (very small to small effect sizes). See Figure 1 for details.

The results of the meta-analysis showed that people with high NA had a tendency to perceive their HGs as being more difficult, less attainable, and less congruent with their identity, whereas people with high PA had a tendency to perceive their HGs as more attainable and more congruent with their identity. The latter was consistent with our hypotheses. The impact of NA on perceptions of HG controllability was very small (*d* = −0.155), and the impact of PA on difficulty was close to zero (0.058). These two results therefore cannot be interpreted as supporting our hypotheses. Moreover, there was an unexpected result—the negative impact of PA on perceptions of controllability.

All the results of our meta-analysis, except for the impact of PA on controllability, had high heterogeneity indices (Q) and must therefore be interpreted with caution. This heterogeneity may be partly related to the fact that the meta-analysis included both state and trait affect. As forest plots D, E, F, and H (Figure 1) clearly show, at least one of the trait affect measures was not aligned with the state affect measures. On the other hand, the heterogeneity may also be due to the impact of moderators, such as emotional intelligence. Further work is needed to assess the impacts of affective states on perceptions of HGs.

## General Discussion

The present studies were designed to further understanding of the observed relationship between NA and health-compromising behaviors. Given that behaviors are guided by goals, we postulated that HGs are instrumental in the adoption and maintenance of health behaviors and that perceptions of HGs are influenced by affect in a mood congruent way. According to Gebhardt (2006), Kruglanski et al. (2015), and Rothermund (2006), people prefer to adopt goals that are easy, controllable, attainable, and congruent with their identity. Consequently, we expected people with high PA to be more optimistic about their HGs and find it easier to pursue them. As a result, they would be more likely to adopt a greater number of health behaviors. In contrast, we expected people with high NA to be more pessimistic about their HGs, find it more difficult to pursue them, and therefore adopt fewer health behaviors.

Our three studies enabled us to assess the relationship between NA and PA and perceptions of HGs. Because affect may be viewed as a trait or a state, we tested the effects of trait affect and state affect on perceptions of HGs. We measured state affect via the PANAS (Studies 2 and 3) and trait affect either via the PANAS (Study 1) or via the neuroticism and extraversion sub-scales of the NEO PI-R (Study 2). Because goals cannot be attained without related means, in Study 3 we were also interested in relationship between state NA and PA and perceptions of HG-related means.

We hypothesized that higher NA would be associated with both HGs and related means being perceived as more difficult and less attainable, controllable, and congruent with self-identity. We expected the opposite pattern from our results for PA. We also expected state affect to be better associated with perceptions of HG than trait affect. Our results supported our hypotheses. The meta-analysis of the results from all three studies showed that participants’ perceptions of HGs were congruent with their affect. Participants with higher NA perceived their HGs as more difficult, less attainable, less controllable, and less congruent with their identity. Conversely, participants with higher PA perceived their HGs as more attainable and more congruent with their identity. PA had a very small influence on perceptions of HG difficulty. However, the impact of PA on perceptions of HG controllability was contrary to our hypothesis, with higher PA associated with lower perceptions of controllability. The HG dimensions regarding which affect had the greatest influence were attainability and congruency with self-identity. Finally, the associations between NA and PA and perceptions of HG-related means were similar to those between NA and PA and perceptions of HGs. Thus, participants with high NA perceived means as less congruent with their self-identity, whereas participants with high PA perceived means as less difficult, more attainable, and more congruent with their self-identity.

To sum up, the results from all three studies suggested that people have tendency to perceive their HGs and related means in mood-congruent way. The associations between perceptions of HGs and affect were stronger for NA than for PA. Our results were consistent with the Feelings-as-information theory (Schwarz and Clore, 1983; Schwarz, 2012), according to which people make judgments in congruency with their mood. Dickson et al. (2011) and Dickson and MacLeod (2006) reported similar results, in their studies participants with clinical depression or depressive symptoms perceived their goals as less controllable.

Unfortunately, our results did not allow us to conclude whether state affect is better associated with perceptions of HGs than trait affect. According to the Feelings-as-information theory, on which our hypotheses were based, people’s judgments are impacted by their current affect when they make the judgment. Several studies of the impacts of state and trait affect, and of anxiety, optimism, and curiosity, have indeed reported that state is a better predictor of decision making outcomes and behaviors (George, 1991; Egloff and Hock, 2001; Mustanski, 2007; Kluemper et al., 2009). Our finding that state PA, but not trait PA, was related to perceptions of HGs is consistent with the Feeling-as-information theory and cited studies. However, our results for state and trait NA were less clear, as both state and trait NA were associated with perceptions of HGs.

Our findings have an important practical implication. As we noted in the introduction, goals that are easy, attainable, controllable, and congruent with an individual’s self-identity are more likely to be adopted and achieved (Gebhardt, 2006; Rothermund, 2006; Kruglanski et al., 2015). Thus, NA appeared to be likely to impede successful HG adoption. Given that NA leads people to believe that their goals and means are less congruent with their identity and makes them view their HGs as more difficult, more stressful, less attainable, and less controllable, we suggest that NA reduces the likelihood that individuals will adhere to their HGs and related means. Moreover, because behaviors are guided by goals (Kruglanski and Köpetz, 2009), people with NA may find it more difficult to adopt and maintain health behaviors. Conversely, PA, which increases perceptions that HGs are attainable and congruent with self-identity, and that means are attainable, congruent with self-identity, and less difficult, is likely to facilitate the adoption and pursuit of HGs and related means. Hence, high PA should facilitate the adoption and maintenance of health behaviors. This is consistent with epidemiological studies, which have shown that people in a negative mood and people with depression have fewer health-promoting behaviors, and that the number of health-compromising behaviors increases with the severity of depression (Allgöwer et al., 2001; Taniguchi et al., 2014; Ferrer et al., 2016).

An important aspect of the present study is that we asked the participants to report their actual HGs. It is important to study self-reported HGs as goals imposed by researchers can be personally irrelevant or difficult to evaluate. For example, non-smokers will not be able to assess attainability or controllability of the HG “quit smoking.” Some critics, however, can be expected in this regard. One can suggest that HG chosen by participants should be rather easy, attainable, controllable, and congruent with their identity. However, some HGs, such as quitting smoking or reducing weight, can be imposed by family members or by a doctor. In this case, the HG may not be linked to self-identity or can be evaluated as difficult or uncontrollable. We believe thus that the design of our study cannot be totally responsible for the obtained results. If this was so, strong associations between affect and all the evaluative dimensions would have been obtained, but this did not occur.

Our studies are correlational, which represents an important limitation. Further work involving experimental or longitudinal studies is needed to confirm the direction of the relationship between affect and perceptions of HGs. It should be noted that Study 1 was underpowered, which could probably explain lack of statistically significant results in this first study. Next, we did not restrain the participants’ choice of HGs because we believe that people are more likely to give accurate responses when evaluating real and important goals. However, this is a potential limitation because the HGs reported by the participants were not equal in terms of difficulty or controllability. For example, stopping smoking or recovering from a serious illness may appear more difficult and less controllable than doing more sport or changing one’s eating habits. Hence, we envisage using a more balanced list of HGs in future studies. Another methodological limitation is related to the fact that we changed the order of presentation of the questionnaires. In Studies 1 and 3, the PANAS was administered after the measure of the HGs, while in Study 2, the PANAS was presented before it. According to several authors (Berkowitz and Troccoli, 1990; McFarland et al., 2003), the fact that participants pay attention to their affect can diminish the effect of affective state on judgment. This hypothesis, however, was not supported by other studies (e.g. White and McFarland, 2009). Further studies are needed to determine whether presentation of measure of affect can bias judgments. Moreover, our approach based on NA and PA can be also a limitation. According to the Appraisal tendency framework (Lerner and Keltner, 2000), specific emotions can have an important influence on judgments and this influence is explained by appraisal tendencies that are unrelated to the valence of affect. This means that emotions of the same valence (e.g. fear and anger) can produce an opposite influence on judgment. We suggest, thus, that the impact of specific emotions should be investigated further in future studies. Finally, the results of the meta-analysis show a small effect size on several evaluation dimensions and a high heterogeneity of results. This can be due to presence of moderators, emotional intelligence, for example. In fact, there is evidence that when people are aware of the source of their emotional state, the impact of this emotional state on judgment is no longer observed (e.g., Schwarz and Clore, 1983). In the present studies, participants with high emotional intelligence may have corrected their appraisal of the HGs in such a way that their affective state exerted little or no effect on these appraisals. Future studies should investigate the role of emotional intelligence.

## Conclusion

Our results suggest that affect is associated with perceptions of HGs and related means in a mood congruent way. The meta-analysis showed the strongest associations between NA and HGs attainability and congruency with self-identity. This relationship suggests that affect, and especially NA, may impact adoption of HGs and subsequently adoption and maintenance of health behaviors. Further studies are needed to determine the direction of this effect and to test possible moderators.

## Data Availability Statement

The datasets generated for this study are available on request to the corresponding author.

## Ethics Statement

All three studies described in the manuscript were carried out in accordance with the recommendations of the code of ethics and University’s guidelines on research integrity with written informed consent from all subjects. All subjects gave written informed consent in accordance with the Declaration of Helsinki. The protocol was approved by the Ethical Commission of the Faculty of Psychology and Educational Sciences.

## Author Contributions

EP and OD planned and designed the studies, designed the perceptions of HGs and related means questionnaires, and translated the PPA and several items of the NEO PI-R into French. EP analyzed the data and wrote the first draft of manuscript. OD revised this draft and approved the final draft for publication.

## Conflict of Interest

The authors declare that the research was conducted in the absence of any commercial or financial relationships that could be construed as a potential conflict of interest.
